# Ability of the rhTSH stimulation test to predict relapse in patients with differentiated thyroid carcinoma, after long-term follow-up

**DOI:** 10.3892/ol.2015.2854

**Published:** 2015-01-07

**Authors:** MAFALDA MARCELINO, ANA FILIPA LOPES, DEOLINDA MADUREIRA, TERESA C. FERREIRA, EDWARD LIMBERT, VALERIANO LEITE

**Affiliations:** 1 Department of Endocrinology, Portugese Institute of Oncology, Lisbon 1600-608, Portugal; 2 Department of Endocrinology, Armed Forces Hospital, Lisbon 1649-020, Portugal; 3 Laboratory of Endocrinology, Portugese Institute of Oncology, Lisbon 1600-608, Portugal; 4 Department of Nuclear-Medicine, Portugese Institute of Oncology, Lisbon 1600-608, Portugal

**Keywords:** recurrence, follow-up, differentiated thyroid carcinoma, rhTSH test, accuracy

## Abstract

The analysis of serum thyroglobulin (Tg) following thyroid-stimulating hormone (TSH) stimulation (sTg) has been recommended in the follow-up of differentiated thyroid carcinoma (DTC) patients, however, its routine use remains controversial. The aim of the current study was to evaluate the accuracy of sTg testing following recombinant human (rh) TSH stimulation in DTC patients, with a follow-up of 12.4 years. Retrospective studies were conducted of 125 DTC patients, who underwent rhTSH stimulation testing between 1999 and 2002. The exclusion criteria were: Patients with anti-Tg antibodies, Tg levels >1 ng/ml under TSH suppression and the absence of radioactive iodine (RAI) ablation therapy following surgery. In total, 49 patients were included in the study and all had been previously treated with total or near total thyroidectomy (with or without central neck dissection) and RAI, postoperatively. The Tg functional sensitivity was 1.0 ng/ml. The follow-up for patients was performed annually. During the median follow-up of 12.4 years after the rhTSH stimulation test, nine patients exhibited recurrence (18.4%). Of the nine patients, six exhibited sTg levels >2 ng/ml (positive result) and three exhibited levels <2 ng/ml (negative result). Relapse occurred at a mean of 5.9 years following the rhTSH stimulation test. The positive predictive value and negative predictive value (NPV) of positive sTg were 50 and 91.9%, respectively, with a sensitivity of 66.6% and a specificity of 85.0%. The rhTSH-stimulated Tg levels have a high NPV, allowing the identification of the patients who are free of the tumour. These results are consistent with the previously published data; however, to the best of our knowledge, this is the study with the longest follow-up duration after rhTSH stimulation.

## Introduction

Thyroid carcinoma comprises 1–2% of all malignancies in the USA (1). Epidemiological studies have shown an increasing prevalence of differentiated thyroid carcinoma (DTC); however the mortality rate has remained stable, most likely due to the increased diagnostic scrutiny (2). Over half of the mortalities in the USA result from papillary carcinoma, the majority of which are low-risk tumours (3). Among DTC patients, ~80% exhibit no evidence of disease following the initial treatment (4). However, the recurrence rate is 10–30% (depending on the initial therapy), and the cancer may reappear after several years (up to one-third of cases recur after the first decade) (5,6), indicating a requirement for prolonged follow-up.

According to the American Thyroid Association recommendations (7), the disease-free status comprises all of the following: i) No clinical evidence of tumour; ii) no imaging evidence of tumour [i.e. no uptake outside the thyroid bed on the initial post-treatment whole-body scan (WBS) or, if uptake outside the thyroid bed is present, no imaging evidence of tumour on a recent diagnostic scan and neck ultrasound (US) is observed]; and iii) undetectable serum thyroglobulin (Tg) levels during thyroid-stimulating hormone (TSH) suppression and stimulation in the absence of interfering antibodies.

The same guidelines also suggest that initial follow-up for low-risk patients should be based predominantly on TSH-suppressive serum Tg (supTg) and neck US, followed by TSH-stimulated serum Tg (sTg) measurements if the supTg is undetectable (7). However, ultrasensitive methods for serum thyroglobulin determination may be used to avoid TSH stimulation 9–12 months following surgery in low-risk patients who have an undetectable serum thyroglobulin on levothyroxine (LT4) treatment (8). This was supported by Brassard *et al* (4), who published a prospective study of 715 DTC patients and concluded that in the majority of patients (84%) who exhibited low supTg levels (<0.27 ng/ml), TSH stimulation did not increase the negative predictive value of Tg determination on the risk of recurrence (99% in the two conditions). In this context, recent publications suggested that negative sTg in the initial approach has such a high negative predictive value, that future sTg are questionable (9,10), and even the initial stimulation test may be replaced by a more sensitive Tg assay (8). Another predictive factor of persistent or recurrent DTC, with high negative predictive value (NPV) and that has been studied in recent years, is the Tg level at the time of recombinant human (rh) TSH-aided ablation, which may be used as a prognostic marker (11).

As the majority of the studies published thus far have a short-term follow-up after the rhTSH stimulation test (≤7 years), and recurrence may occur in up to one-third of cases after the first decade, the present study aimed to evaluate the accuracy of rhTSH-stimulated Tg levels in patients with undetectable supTg values (on LT4 therapy), to predict the remission after a follow-up of 12.4 years.

## Patients and methods

### Patient selection

Between 1999 and 2002, the determination of Tg levels following rhTSH stimulation testing was routinely performed in low-risk DTC patients at the Department of Endocrinology at the Portugese Institute of Oncology (Lisbon, Portugal). This study was approved by the ethics committee of the Portugese Institute of Oncology and written informed consent was obtained from all patients.

**Figure 1:**
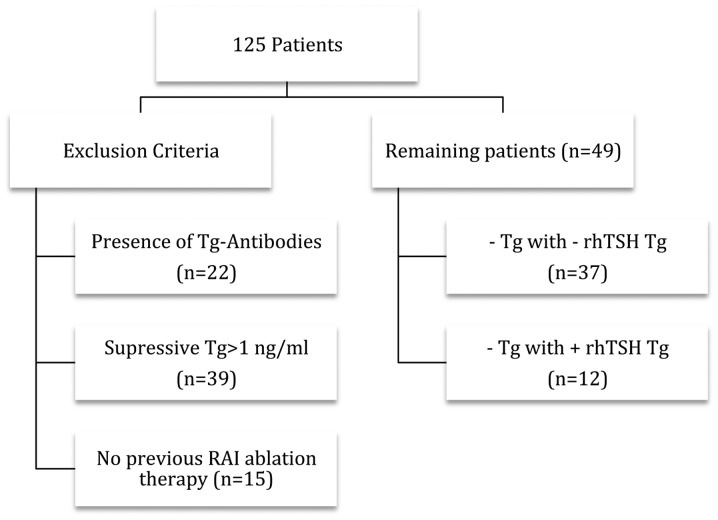
Flowchart for patients with application of exclusion criteria. Tg, thyroglobulin; rhTSH, recombinant human thyroid-stimulating hormone; RAI, radioactive iodine.

In total, 125 DTC patients were evaluated who underwent a total or near total thyroidectomy, which was completed by central neck dissection in 38% of cases, resulting in the apparent complete resection of the neoplastic tissue. Overall, 88% of these patients underwent radioactive iodine (RAI) ablation therapy postoperatively. For all but one patient, no focus of uptake outside the thyroid bed was detected on the WBS, performed 2 days following ^131^I therapy. Subsequently, all patients were treated with suppressive LT4 doses. Tumour stages were classified according to the TNM scoring system (12). Exclusion criteria for this study were: i) presence of anti-Tg antibodies; ii) TSH-suppressive Tg levels >1 ng/ml; and iii) absence of RAI ablation therapy following surgery. Following the application of these criteria, 49 patients remained in the study (Fig. 1), who were divided in two groups according to: i) Positive sTg levels >2 ng/ml and ii) negative sTg levels <2 ng/ml.

### Procedures

Patients were given 0.9 mg rhTSH intramuscularly on two consecutive days, followed by 4 mCi ^131^I after 24 h. Additionally, the serum Tg measurement and diagnostic WBS (DxWBS) were conducted on day 5 (72 h following the final rhTSH injection).

DxWBS was classified as follows: i) negative when no uptake was observed or in the case of neck iodine uptake consistent with a normal thyroid remnant; or ii) suspicious in the case of uptake outside the thyroid bed, suggesting underlying disease. The Tg measurements were obtained using manual immunoradiometric Tg assays, (SELco TG kits; Medipan GmbH, Selchow, Germany) with a functional sensitivity of 1 ng/ml. Based on the functional sensitivity of the assay, a concentration of 1 ng/ml was selected as the cut-off value discriminating undetectable from detectable Tg levels.

### Follow-up

Follow-up assessments were conducted annually and consisted of a clinical examination, serum supTg determination under LT4 treatment and occasionally, neck US. Patients were categorised as ‘no evidence of disease’ (NED) if the neck US was negative and if all supTg levels were <1.0 ng/ml. Persistent tumours were identified by fine needle aspiration cytology (when the neck US was positive) or by ^131I^ WBS showing ^131^I uptake outside the thyroid bed. Chest computed tomography was performed when the neck US was positive or when the supTg levels were >1.0 ng/ml with negative US.

### Statistical analysis

Statistical analysis was performed using SPSS, version 20 (IBM, Armonk, NY, USA). Comparisons of the categorical data were performed using the χ^2^ test (Fisher’s exact). P<0.05 was considered to indicate a statistically significant difference. The results of the 49 patients were analysed for estimation of the accuracy of the rhTSH stimulating Tg levels. A 2×2 analysis was constructed of NED (yes or no) and this was compared to sTg levels (negative or positive with a cut-off value of >2.0 ng/ml). The stimulated Tg accuracy was assessed through sensitivity, specificity, positive predictive value (PPV) and NPV determinations.

## Results

### Population data

The clinical characteristics of the study population are reported in Table I. All patients received at least one ablation dose of ^131^I, nine patients (18.8%) received two doses, and nine received three or more doses, with a mean dose of 160 mCi (range, 50–670 mCi). The tumour stages are shown in Table I. Distant metastases were observed in one patient when the initial cancer diagnosis was determined; however, an excellent response to therapy was exhibited, resulting in negative ^131^I WBS and serum supTg levels <1.0 ng/ml, and the patient was considered free of disease at the time of sTg evaluation. The time between initial surgery and the rhTSH stimulation test ranged from 1–36 years [mean ±standard deviation (SD), 7.9± 7.6 years].

### Follow-up and recurrence

The mean (±SD) follow-up after the rhTSH stimulation test was 12.4 years (± 0.7 years), with a minimum of 10 years and a maximum of 13 years. In total, eight of the 49 patients were lost to follow-up, seven patients exhibited no evidence of disease and one exhibited recurrent disease at the time of the last appointment (5 years following the rhTSH stimulation test). Following the rhTSH stimulation test, 75.5% (37/49) of the patients with baseline Tg levels <1.0 ng/ml exhibited no increase in sTg levels (<2 ng/ml). By contrast, 12 of the 49 patients (24.5%) exhibited rhTSH-stimulated Tg levels >2 ng/ml. DxWBS following rhTSH administration was negative in 23 patients (46.9%), revealed uptake in the thyroid bed in seven patients (14.3%) and was not performed in 19 patients (38.8%).

### Stimulated Tg accuracy

In total, nine recurrences (18.4% of patients) were identified during a mean of 12.4 years of follow-up after the rhTSH stimulation test. Recurrence was detected after a median (±SD) of 5.9 years (± 2.0 years). These recurrences occurred in the thyroid bed in two cases (22.2%), in the lateral neck lymph nodes in three cases (30%), and in the lungs and bone in one case each (11.1%). Additionally, two cases (22.2%) with persistent elevated supTg levels were identified; however, no imaging or clinical evidence of disease was observed. All recurrences occurred in patients with papillary cancer and all of the patients exhibited lymph node metastasis (N1) at presentation. Of all the 49 patients, 22 had lymph node metastasis and 40.9% of these had a recurrence. The individual relapse time is shown in Tables II and III.

Among the 12 patients with sTg levels >2 ng/ml, only six patients experienced a recurrence [true positive (TP)], leading to a PPV of 50%. Among the other 37 patients who exhibited negative sTg levels (<2 ng/ml), 34 patients did not experience any recurrence (true negative (TN)], leading to an NPV of 91.9%. Three false negatives and six false positives (Table IV) were also identified. Analysing this data, a sensitivity of 66.6% and a specificity of 85.0% was estimated for the sTg levels.

## Discussion

The upgrading of Tg assays has been associated with an improvement in diagnostic accuracy, leading to a lower prevalence of tumour recurrence than in the past, among patients declared free of disease (13). In the current retrospective study, the aim was to evaluate the accuracy of Tg-stimulated levels and its NPV and PPV in 49 patients subjected to total thyroidectomy and RAI ablation therapy with a long follow-up duration. A sensitivity of 66.6% and a specificity of 85% were obtained, with a PPV of 50% and an NPV of 91.9%.

As described in a number of previous studies (13–15), rhTSH sTg may have a low sensitivity and low PPV. Results have been published indicating that ~20% of patients who are clinically free of disease, with serum Tg levels <1 ng/ml during suppression of TSH, are likely to have a serum Tg levels >2 ng/ml after rhTSH or thyroid hormone withdrawal at 12 months following surgery and RAI. In these patients, only one-third are likely to have an indication of persistent or recurrent disease and increasing Tg levels (7). Due to this, sTg may result in unnecessary additional testing and treatment in a number of patients. In the current study, only half of the patients with sTg levels >2 ng/ml exhibited evidence of disease in the follow-up. Conversely, the NPV was high (91.9%), allowing the identification of patients unlikely to experience disease recurrence and permitting less aggressive management strategies. These results are consistent with previously published results (Table V) (13).

In recent years, several studies have compared ultrasensitive Tg assays with sTg, suggesting similar NPVs (99 vs. 100%) (16,17). In low-risk patients, it was suggested that TSH stimulation tests may be avoided when sensitive suppressive Tg levels are low (<0.27 ng/ml). In this study, rhTSH stimulation improved the positive predictive value of serum sTg determination only in patients with supTg levels >0.27 ng/ml, and the authors hypothesised that rhTSH must be restricted to such patients (4,8). However, several studies using Tg assays with lower functional sensitivities have demonstrated that improved Tg sensitivity for disease detection is counterbalanced by an increase in false positives (17,18).

When considering the DTC recurrence rate, long-term follow-up may demonstrate higher rates of tumour recurrence (13). This hypothesis was also described by Mazzaferi and Kloos (6), who reported locoregional recurrences and distant metastases (15 and 20%, respectively) more than two decades following the initial therapy. The long follow-up period (12.4 years) is a significant factor in the current report, and allowed the confirmation that the recurrence rate remains high (18.4%), even in a group of patients considered to be free of disease. Comparing this with the results published to date (Table V), a higher relapse rate was observed in the current study, which is likely to be due to the duration of follow-up, which was significantly longer than in the compared studies. In the current study, almost all the patients with recurrent disease were in TNM stage I (88.9%) predominantly due to age (<45 years). The classification systems that use age to stratify risk may be inaccurate in predicting recurrence-free survival, as young patients have high recurrence rates (6). However, all patients with recurrences exhibited lymph node metastases (N1) at initial surgery, which is a well described risk factor for recurrent disease (6,19). In the current study, the recurrence rate in patients with lymph node metastases was 40.9%.

Pacini *et al* (20) suggested that neck US combined with sTg has the highest diagnostic accuracy for the detection of persistent disease, without the requirement for diagnostic WBS. In the current study, although 38% of the relapsed patients had not undergone DxWBS following rhTSH administration, a large number of false negatives in DxWBS were identified, leading to a low sensitivity and a low PPV of this diagnostic approach, confirming the results from other groups (21).

In the majority of published studies, Tg stimulation tests are performed within 1–2 years of surgery and ^131^I, considering that the majority of recurrences occur early following the primary treatment. By contrast, the current study includes patients whose primary treatment occurred within varied and occasionally long periods prior to Tg stimulation tests (median, 8.6 years; range, 1–36 years). Additionally, this study confirmed that late recurrences may occur in differentiated thyroid cancers, indicating that, even when varied and long periods of time following primary treatment have elapsed, the prognostic ability of the rhTSH stimulation test, particularly its NPV, is maintained.

In conclusion, the accurate surveillance for possible recurrence in patients considered to be free of disease is the predominant goal of long-term follow-up. With new sensitive serum Tg assays, rhTSH stimulation tests may not be routinely necessary. The benefit is greater in patients who have undetectable sTg levels (without serum Tg antibodies), allowing the identification of the patients who are free of disease. In these patients it is possible to ensure a more cost-effective and safe follow-up, monitoring supTg levels and performing neck ultrasound on an annual basis.

## Figures and Tables

**Table I:** Characteristics of the 49 patients at presentation.

	Patients (n=49)	
	
Characteristics	n	%
Gender		
Female	41	83.7
Male	8	16.3
Mean age, years	60.6	
TNM classification (12)		
T1	4	8.2
T2	10	20.4
T3	9	18.4
T4	7	14.3
Tx	19	38.8
Lymph nodes		
N0	21	42.9
N1	22	44.9
Nx	6	12.2
Distant metastasis		
M0	48	98.0
M1	1	2.0
Stage		
I	28	57.1
II	6	12.2
III	5	10.2
IV	1	2.0
Unknown	9	18.4
Histology		
Papillary	44	89.8
Follicular	4	8.2
Papillary + follicular	1	2.0
Surgery type		
Total thyroidectomy	24	49.0
Near total thyroidectomy	6	12.2
Total thyroidectomy with central neck dissection	19	38.8

**Table II:** Recurrent disease in negatively stimulated thyroglobulin (<2 ng/ml) patients: False negative.

Gender, age^a^ (years)	Surgery	Histology	TNM	^131^I total dose, mCi	^131^I treatments, n	DxWBS	sTg, ng/dl	Recurrence	Time of recurrence Cir/sTg, years
F, 31	TT + CND	Papillary	T3mN1M0	130	2	Neck uptake	<1	AATg^+^ Lung	21/7
F, 39	TT + CND	Papillary	T2mN1M0	220	2	Negative	<1	Tg ↑ thyroid bed	10/6
F, 49	TT + CND	Papillary	TxN1M0	163	2	Neck uptake	<1	Tg ↑ Bone	26/3

a Age at the time of initial therapy of thyroid cancer.

F, Female; M, Male; TT, total thyroidectomy; CND, central neck dissection; DxWBS, diagnostic whole body scan; sTg, thyroid-stimulating hormone-stimulated serum thyroglobulin; Cir, surgery.

**Table III:** Recurrent disease in positively stimulated thyroglobulin (>2 ng/ml) patients: True positive.

Gender, age^a^ (years)	Surgery	Histology	TNM	Stage	^131^I total dose, mCi	^131^I treatments, n	DxWBS	sTg, ng/dl	Recurrence	Time of recurrence Cir/sTg, years
M, 43	TT + CND	Papillary	TxN1M0	I	184	2	Negative	2.7	Tg ↑ Lymph nodes	19/6
F, 41	TT + CND	Papillary	TxN1M0	I	50	1	Neck uptake	3.2	Tg ↑ Lymph nodes	19/10
M, 24	TT + CND	Papillary	T2N1Mx	I	278	3	Negative	2.6	Tg ↑ Thyroid bed	13/5
F, 22	TT + CND	Papillary	T4N1M0	I	502	5	Neck uptake	35.9	Tg ↑ No imag.	8/5
F, 32	TT + CND	Papillary	T3N1M0	I	561	4	Neck uptake	9.1	Tg ↑ Lymph nodes	7/4
F, 40	TT	Papillary	T2N1M0	I	70	1	Neck uptake	2.8	Tg ↑ No image	8/7

a Age at the time of initial therapy of thyroid cancer.

F, Female; M, Male; TT, total thyroidectomy; CND, central neck dissection; DxWBS, diagnostic whole body scan; sTg, thyroid-stimulating hormone-stimulated serum thyroglobulin; Cir, surgery.

**Table IV:** Crosstable between NED and sTg level.

	NED		
		
	Yes	No	Total
sTg, ng/ml			
<2	34 (TN)	3 (FN)	37
>2	6 (FP)	6 (TP)	12
Total	40	9	P=0.001

NED, non evidence disease; sTg, thyroid-stimulating hormone (TSH)-stimulated serum thyroglobulin; TN, true negative; FN, false negative; FP, false positive; TP, true positive.

**Table V:** Comparison between studies of accuracy of sTg levels following recombinant human TSH stimulation.

First author (year) [ref.]	Mean follow-up time after sTg test, years	Time of sTg test, years	sTg cut-off (ng/ml)	Recurrence rate, %	Sensitivity, %	Specificity, %	Positive predictive value, %	Negative predictive value, %
Present study	12.4	7.9	>2.0	18.4	66.6	85.0	50.0	91.9
Brassard (2011) [4]	6.2	0.75–1.0	>1.4	4.5	78.0	90.0	26.0	99
Kloos (2010) [13]	7	5.5	>2.5	17	80	97	84	95
Robbins (2002) [14]	2	<2	>2.0	15.5	56.3	88.5	47.4	91.7
Schlumberger (2007) [18]	2.33	0.75–1.0	>0.9	<3	68–76	81–91	NR	NR

sTg, TSH-stimulated serum thyroglobulin; TSH, thyroid-stimulating hormone; NR, not recorded.
